# A Case Study of a Whole System Approach to Improvement in an Acute Hospital Setting

**DOI:** 10.3390/ijerph19031246

**Published:** 2022-01-22

**Authors:** Marie E. Ward, Ailish Daly, Martin McNamara, Suzanne Garvey, Sean Paul Teeling

**Affiliations:** 1 Centre for Innovative Human Systems, School of Psychology, Trinity College, The University of Dublin, D02 PN40 Dublin, Ireland; marie.ward@tcd.ie; 2 Beacon Hospital, Sandyford, D18 AK68 Dublin, Ireland; Suzanne.garvey@beaconhospital.ie; 3 UCD Centre for Interdisciplinary Research, Education & Innovation in Health Systems, School of Nursing, Midwifery & Health Systems, UCD Health Sciences Centre, University College Dublin, D04 V1W8 Dublin, Ireland; martin.mcnamara@ucd.ie (M.M.); sean.p.teeling@ucd.ie (S.P.T.); 4 Centre for Person-Centred Practice Research Division of Nursing, School of Health Sciences, Queen Margaret University, Queen Margaret University Drive, Musselburgh EH21 6UU, UK

**Keywords:** whole system improvement, socio-technical systems, Lean Six Sigma, person-centred care, acute hospital, implementation science

## Abstract

Changes in healthcare tend to be project-based with whole system change, which acknowledges the interconnectedness of socio-technical factors, not the norm. This paper attempts to address the question of whole system change posed by the special issue and brings together other research presented in this special issue. A case study approach was adopted to understand the deployment of a whole system change in the acute hospital setting along four dimensions of a socio-technical systems framework: culture, system functioning, action, and sense-making. The case study demonstrates evidence of whole system improvement. The approach to change was co-designed by staff and management, projects involving staff from all specialities and levels of seniority were linked to each other and to the strategic objectives of the organisation, and learnings from first-generation projects have been passed to second and third-generation process improvements. The socio-technical systems framework was used retrospectively to assess the system change but could also be used prospectively to help healthcare organisations develop approaches to whole system improvement.

## 1. Introduction

The Patient Safety and Quality Improvement (QI) movements in healthcare have been slow to achieve momentum in improving outcomes [1]. Braithwaite et al. (2018) estimate that in healthcare organisations, nearly two-thirds of initiatives experience implementation failure [2]. Changes in healthcare tend to be project-based with whole system change, which acknowledges the interconnectedness of socio-technical factors, not the norm. In addition, it can be difficult both to sustain change beyond the project lifecycle as well as to generalise change to a broader level [3].

Lean Six Sigma is a powerful methodology that reduces waste and variation in an organisation and ultimately minimises operating costs, optimises productivity, and maximises customer satisfaction [4]. LSS is the merger of two methods used in process improvements. Lean originated in Toyota car production factories and focuses on refining and improving processes as well as eliminating non-value-added (NVA) activities [5]. Six Sigma was introduced by Motorola to optimise its manufacturing processes by reducing their variability through the rigorous application of process metrics collection and statistical analysis [6,7]. Since the early 2000s, LSS thinking has been adapted into healthcare with the goal of improving patient safety, quality of care, efficiency, patient satisfaction, and performance [8].

Healthcare providers worldwide, both publicly and privately funded, are faced with similar challenges of caring for an ageing population with a limited pool of financial and personnel resources. Consequently, the need to seek improved efficiencies while continuing to provide safe and high-quality services has become more and more acute [9]. LSS has been implemented in many healthcare organisations, with impacts achieved across many clinical and administrative pathways and processes [10,11]. While there are positive associations between LSS adoption and performance indicators in individual case studies [12,13,14,15], overall evidence on the success of LSS is mixed. Considerable time and effort need to be spent on implementation for LSS to be associated with gains in hospital performance. The degree to which this investment is made depends on the system maturity, leadership commitment, daily management system use, and training [16,17]. There is also increasing recognition of the importance of improving both patient and staff experience of healthcare [18,19] and moving to person-centred approaches in healthcare [20]. Political and policy stakeholders have widely advocated that person-centred care should be at the heart of the health system [21,22,23,24]. Person-centredness refers to embedded practices within a specific type of culture that enable and facilitate the delivery of person-centred care [25,26]. Person-centred cultures are deemed necessary for the delivery of person-centred care [26]. Person-centred care has an explicit focus on ensuring that the client or patient is at the centre of care delivery [25,27] and is concerned with every person involved in the patient’s care, including staff members and patients and their families/carers [20,27].

Implementation science as a field aims to help understand the factors surrounding the uptake of evidence-based practice into healthcare [28]. A central tenet of implementation science is that implementation strategies will be most successful when they align with healthcare systems’ existing culture, infrastructure, and practices [29]. Context has thus emerged as a key construct in understanding challenges to healthcare improvement [30]. Inconsistencies exist, however, in defining context [31] and in understanding the complexity of context in healthcare [32].

When talking about the healthcare system as a whole system, it is important to refer to a method for describing such a system that addresses its complexity and provides an analysis that gives leverage over the mechanisms of system change. McDonald et al.’s 2021 [33] work presented in this special issue makes a cogent argument for the importance of taking a socio-technical systems (STS) approach to whole system understanding and change. STS analysis involves studying the dynamic interconnectedness of elements of the system at different levels, such as team, processes, and information and knowledge. They propose a model called the CUBE for STS analysis that focuses on four domains:

### 1.1. Culture

Culture represents the pattern of shared basic assumptions and (what is often) a partial shared understanding of the STS and incorporates Schein’s [34,35] and Pigeon and O’Leary’s [36,37] work on culture.

### 1.2. System Functioning

System functioning represents how the system actually works and incorporates both formal elements (work-as-imagined), i.e., Policies, Procedures, Protocols, and Guidelines (PPPGs) as well as informal elements (work-as-done or the sequence of activities that normally takes place) [38] and incorporates Perrow’s functional focus on complexity and coupling [34].

### 1.3. Action

Action represents how we act within the system, incorporates Turner and Pidgeon’s work on the flows of information, knowledge and understanding, and anything that happens in the system that is recordable or measurable [37]; this can be analysed at different levels, such as individual actions, team performance against a standard, activity sequences, or key outcome, process, and balancing measures in relation to system performance [35].

### 1.4. Sense-Making

Sense-making represents how we understand and make sense of our world and incorporates Weick’s work on how individuals operating within the system make sense of it, often through practical action [39].

**Figure 1:**
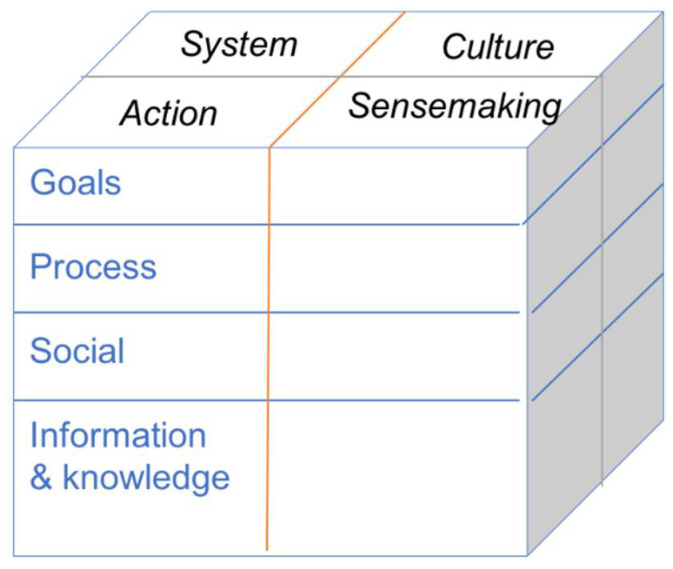
Pictorial representation of the CUBE.

These dimensions of the CUBE are further broken down in terms of four types of relation: Goals (linked to objectives and outcomes), Process (sequential relations), Social Relations (reciprocal relations of working with and reporting to), and Information and Knowledge (exchanges of meaning that link people and processes). Figure 1 represents the CUBE.

This case study reports on the system-wide implementation of LSS in conjunction with person-centred care principles in a large acute private hospital setting. The organisation’s mission is to provide exceptional patient care in an environment where quality, respect, caring, and compassion are central. This mission is based on organisational values of dignity, excellence, collegiality, and communication. In 2014, the organisation set out on a journey of expansion and growth. It was recognised that if this was to be achieved while holding the highest standards in quality and safety of patient care all staff would need to be involved and play a role. At that time, the organisation had achieved accreditation by the Joint Commission International and to maintain this was a key organisational goal [40].

This case study sets out to address the question ‘Was the deployment of LSS and person-centred care in this hospital a change on a whole system level?’. The CUBE will be employed as a descriptive and analytic framework to help answer this question.

The CUBE framework is firstly used here to outline some of the important considerations prior to the commencement of the change programme.

### 1.5. Culture

There was a recognition of the importance of culture from the outset. Retention and development of a highly-skilled staff body with significant organisational knowledge would be crucial to the journey of expansion. A key organisational priority became adopting a person-centred approach with the principles of collaboration, inclusiveness, and participation (CIP) underpinning process improvement in the hospital [20].

### 1.6. System

The following strategic objectives were set in 2014: to ensure excellence in quality and safety of patient care through compliance with the six International Patient Safety Goals as outlined by Joint Commission International [35]; to use Information Technology to enhance Safer Patient Care; to improve Patient Flow, and to improve Care of the High-Risk Patient. With the setting of these strategic objectives, it was recognised that all improvement work needed to come under one approach and be aligned to these strategic objectives as set out in the Hospital Leadership Goals 2014 [41]. This has been a criticism of QI in healthcare with the term ‘projectitis’ referring to an excessive focus on small projects that are not aligned to the strategic goals of the organisation or each other [42].

### 1.7. Action

Not all action in healthcare is suitable for easy measurement. A key focus of the hospital’s efforts, however, would be the ability to measure current performance and to know when a change is an improvement [43,44]. Another priority would be to give healthcare teams information and knowledge on how they were performing so that they would make sense of their own processes and improvement [38,45].

### 1.8. Sense-Making

Providing staff with excellent educational and developmental opportunities would be essential to support sense-making. The desired “future state” was a better patient and staff experience supported by a culture where all staff members, from Board and Executive Management Team (EMT) to frontline staff, had a shared vision of the goals and adopted a system-wide approach to process improvement, avoiding working in silos [46]. The organisation had a strong history of supporting staff in the completion of post-graduate education and training; however, before this project, education and training opportunities had been considered based on the individual’s or possibly the department’s needs. Outputs were delivered at the individual or departmental level. A system-wide consideration of education and training needs and outputs had not previously been attempted. It would be essential that staff were educated together to achieve a system-wide approach to change and improvement.

## 2. Materials and Methods

### 2.1. Case Study

A case study approach [47,48] was adopted here to understand the deployment of a whole system change in the acute hospital along the four dimensions of STS outlined above. A case study is an approach that is used to generate an in-depth, multi-faceted understanding of a complex issue in its real-life context [49]. This case study sets out to address the question ‘Was the deployment of LSS and person-centred care in this hospital a change on a whole system level?’. The case study analysis was informed by a number of different sources of evidence [47].

### 2.2. Evidence

#### 2.2.1. Internal Hospital Documentation

Hospital Leadership Goals (2014)

Education and Training Working Group; agendas and minutes (2015–2021)

Education and Training Working Group; gap analysis (2015)

Lean Academy presentation to the Hospital Board of Directors (2016)

LSS projects; meeting notes, project progress tracking (2017–2021)

#### 2.2.2. Seven Research Studies Presented in This Special Issue

Operation Note Transformation: The Application of Lean Six Sigma to Improve the Process of Documenting the Operation Note in a Private Hospital Setting [50].

Releasing Operating Room Nursing Time to Care through the Reduction of Surgical Case Preparation Time: A Lean Six Sigma Pilot Study [51].

Redesigning the Process for Scheduling Elective Orthopaedic Surgery: A Combined Lean Six Sigma and Person-Centred Approach [52].

Lean Six Sigma Redesign of a Process for Healthcare Mandatory Education in Basic Life Support—A Pilot Study [53].

The Use of Lean Six Sigma for Improving Availability of and Access to Emergency Department Data to Facilitate Patient Flow [54].

Using Lean Six Sigma to Redesign the Supply Chain to the Operating Room Department of a Private Hospital to Reduce Associated Costs and Release Nursing Time to Care [55].

The Use of Lean Six Sigma Methodology in Reducing Length of Stay and Improving Patient Pathway in Anterior Cruciate Ligament Reconstruction Surgery (submitted) [56].

#### 2.2.3. Participant Observation

One of the authors (AD) is the Director of Education, Innovation, and Rehabilitation at the hospital and has been on this whole system change journey since 2014. She has observed most of the processes concerning the deployment of LSS and person-centred care across the hospital. Another author participated in the Education and Training Working Group (SG). Another author (SPT) is one of the staff members from the Lean Academy who has also been involved since the beginning of the deployment from an academic provision perspective and has observed the system change unfold through this lens since 2017.

### 2.3. Synthesis

The synthesis of the evidence was facilitated by two authors (MEW and MMcN). MEW was involved in the development of the STS CUBE framework [33,57] and MMcN developed the university-accredited LSS curriculum to overcome system blindness [58], which was used within the hospital. MEW and MMcN supported the synthesis of the evidence by using questions from the CUBE framework combined with reflective questions from Oshry’s Organic Systems Framework (OSF) [59,60]. Because of the participatory nature of the involvement, it was felt important to add this reflective dimension. Oshry’s OSF provides a framework and vocabulary for describing human systems as organic wholes and allows us to understand and, potentially, influence a range of system phenomena. Oshry’s concepts enable us to see the whole as a pattern of systemic relationships (what the whole is) and as a pattern of systemic processes (what the whole does). He addresses how, as system members, we experience ourselves, our relationships with others, the systems we are a part of, other systems, and the relationships among systems, and it allows us to make more informed decisions and to take more informed actions based on these experiences. A set of questions based on the CUBE and Oshry’s OSF can be found in Table 1 and Table 2. These questions were posed by MEW and MMcN to the other authors and answered through a process of iteratively writing up this case study. The synthesis set out to generate an answer to the question of whether or not this change could be described as being at a whole system level.

### 2.4. Approach to Change

The approach to change at the time of commencement is now outlined under the domains of the CUBE.

#### 2.4.1. Culture

Simpson et al. (2019) describe the importance of healthcare organisational culture when considering quality and patient safety in healthcare [49]. In 2014, the organisation culture was evolving from a “Power Culture” where the key to the organisation sits in the centre surrounded by widening circles of intimates and influence [61] (Handy 1999 p. 86). While such a command-and-control culture supported the successful initial drive to build and open the hospital, there was an acknowledgement that a challenge to sustaining and developing an organisation based on a “Power Culture” can be high staff turnover and staff dissatisfaction. There was a need to evolve to a culture of collaboration, inclusion, and participation, allowing the right staff power and influence to contribute to service progression and ultimately organisational development and expansion [20].

#### 2.4.2. System

The strategic goals that the change was to support are outlined in Table 3. These are aligned to the JCI accreditation program chapters. JCI accreditation had been achieved by the organisation and a key strategic goal was to maintain this accreditation.

#### 2.4.3. Action

Each part of the change process would address a strategic goal and would need to achieve certain pre-defined outcomes as outlined in Table 4 below.

#### 2.4.4. Sense-Making

With support from the Board of Directors and the EMT, an Education and Training Working Group (ETWG) was created to identify the needs of the organisation and recommend relevant education and training programmes for implementation. The ETWG comprised a diverse set of stakeholders, all with a crucial role in developing a strategic direction for the organisation. The ETWG agreed on the importance of including all staff in opportunities to input into the design of the education programme; however, they also identified the challenge in accessing and meeting with a wide number of staff productively and effectively. Therefore, an open platform for suggestions was created through town hall meetings, departmental meetings, and performance reviews, including training needs analysis. Each ETWG member took responsibility for a staff/departmental grouping to gain their thoughts on education and training requirements as outlined in Table 5.

Engagement sessions were structured as focus groups with one-to-one sessions also facilitated when requested. The results of the stakeholder engagement sessions helped to inform the desired outcome of education and training solutions as outlined in Table 6.

Participants were asked to consider focus group themes in the context of the wider organisation rather than discipline or department-specific and the context of the deliverables outlined by the hospital Board of Directors and EMT. To ensure inclusion, a representative from all departments was invited to contribute. When choosing a representative, departments were encouraged to consider staff from all grades/groupings—not specifically managers.

Based on feedback from stakeholders, the ETWG proceeded to scope potential education and training solutions with some key outcomes required in the following areas:

- the culture of quality and patient safety as a priority goal for the organisation would need to be endorsed in any education and training programme;
- to continue to deliver the best patient care, the organisation would need to constantly evolve and improve, working to best international evidence-based practice; and
- the programme would need to take account of the strategic direction of the organisation, including the use of technology to enhance patient care, optimise patient flow, and optimise care of the high-risk patient.

The ETWG identified that the gap in organisational knowledge lay not in the theory of what care to provide but the project management and process improvement skills to bring those theories to fruition. Rather than middle management/senior clinicians passing an idea to EMT to realise, the goal was to achieve a system-wide change in how projects are delivered—co-creating and realising strategies with senior and middle management and frontline staff working together [62]. Thus, education and training would need to be accessible to team members from all disciplines and all levels of seniority. To support future goals of improved inter-professional collaborative and shared decision-making, education and training that was accessible to the wider healthcare team across levels of seniority, from EMT to department managers as well as staff directly involved in the patients’ journey through the organisation, was deemed a priority [62].

To add accountability to students and the organisation, a formal academic qualification was deemed a requirement. This was to ensure that students would receive official recognition of knowledge gained and the organisation would be able to formally identify deliverables from investment in training that could be expected.

With education requirements defined (Table 6), the ETWG completed a scoping review of literature of Cinahl and PUBMED databases using keywords including Process Improvement, Healthcare, and Person-Centredness. Emerging evidence of the role of LSS in wider healthcare settings was identified. Of particular note was the variation in LSS work completed in healthcare settings, including administration/patient scheduling, Emergency Department patient flow, Theatre flow, and laboratory turnaround times [11,63,64,65,66], as well as the impact of LSS in improving quality, patient safety, and employee engagement in healthcare [27]. The ETWG identified LSS as an evidence-based approach to process improvement. Its background in business and then healthcare aligned with the logistics of merging clinical and business process improvements in a private healthcare setting. The principles of LSS include recognising the complexity of healthcare, avoiding silo working, always being open to change and improvement, gathering data to create knowledge, cutting waste not care and focusing on improving the process rather than seeking person-specific improvements that matched the ethos of the organisation.

The ETWG took the evidence from the literature and sought further information regarding the impact of LSS in healthcare through visiting sites that had successfully implemented LSS to examine the “lived experience” of the organisation and their team. This took the form of a site visit to an acute hospital as well as attendance at a White Belt: “Fundamentals of Process Improvement for Healthcare” provided by the Mater Lean Academy. On assessing the literature and reflecting on the site visit, the ETWG reflected on the potential for LSS in healthcare as an education and training resource for process improvement in the organisation. The specific advantages related to accessibility. The structured delivery of LSS from White Belt: “Fundamentals of Process Improvement for Healthcare” to Green Belt: “Professional Certificate Process Improvement in Health Systems” to Black Belt: “Graduate Diploma Process Improvement in Health Systems” would enable staff at all levels to access LSS training—from a 1-day training course to a 1-year diploma.

The ETWG agreed to recommend LSS as an education programme to support process improvement in the organisation. The hospital Board of Directors supported the recommendation and an implementation plan was agreed upon. The support of the Board and EMT was a key requirement before the implementation plan and was based on the following principles:

- LSS training would be made available to all staff. Training would not be discipline or grade-specific. This was important in developing staff who ‘can’, contextualising the change across the organisation, and recognising the role of all employees [62].
- The method of delivery would be the same for all staff—thus, there was no specific delivery methodology for the EMT.
- The organisation would fully support participation in LSS education events. This included the provision of study leave and financial support for attendance at LSS training events. Thus, the improvement approach was resourced from the outset.
- Members of the EMT were committed to attending training events and acting as executive sponsors as projects emerged. This confirmed leadership commitment through walking the walk, getting involved, and supporting the project [50,51,52,53,54,55,56].

## 3. Results

### 3.1. How Change Was Achieved in the Organisation

The details for how each individual project achieved its goals are written up in the accompanying papers to this case study [50,51,52,53,54,55,56]. Some examples of quality and patient safety improvement include: a reduction in the length of stay for surgeries, leading to less likelihood of acquiring a healthcare-associated infection; an increase in capacity to deliver Basic Life Support across the organisation; surgical notes transferred to electronic platforms to improve legibility and accessibility; and releasing nursing and healthcare assistants time to care for patients. Please see Table 7 for a full list of outcomes.

The mechanisms for change at a system level are presented here using the four domains of the CUBE.

#### 3.1.1. Culture

As can be seen in Table 7, it is evident that the teams involved in the process improvement projects were from a wide range of backgrounds and seniority, some directly involved in the process, some giving an external perspective. Working from a common framework of the LSS methodology underpinned by a person-centred approach has allowed voices across disciplines and seniority to take an active role in project delivery. It has allowed for devolved responsibility for project delivery from the EMT level. The organisational culture shifted from a power-based culture to a task-based culture [61].

#### 3.1.2. System

**Figure 2:**
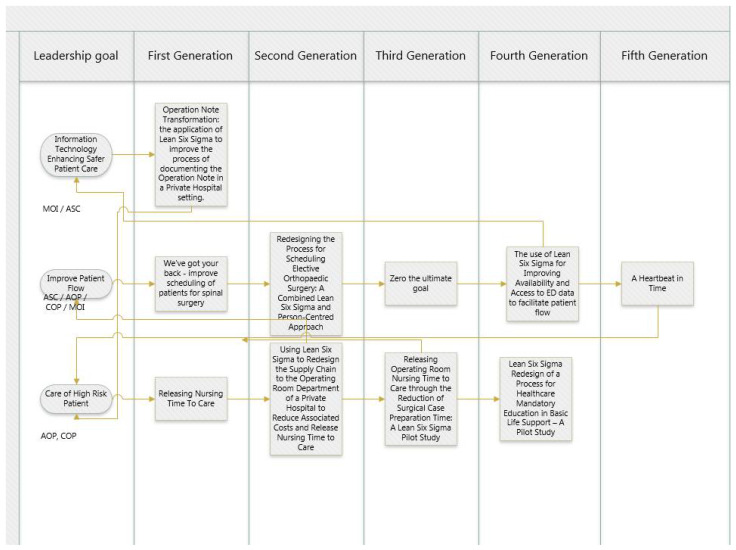
Interconnection of projects supporting multiple strategic targets.

All projects supported organisational strategic goals as well as quality and patient safety priorities. Table 7 demonstrates the system-wide impact of process improvement projects delivered to date. Learnings from first-generation projects have been passed to second and third-generation process improvements (Figure 2). Rather than being completed in isolation, projects are linked and outcomes are used to inform further process improvement.

#### 3.1.3. Action

Each of the projects described in Table 7 has resulted in concrete tangible outcomes for the organisation. For example, the Emergency Department data are circulated daily to the Emergency Department and EMT [54]. The use of LSS to redesign the delivery of Basic Life Support (BLS) training has resulted in a 50% increase in the capacity to deliver BLS [53]. Key to this was the academic qualification attached to the LSS training. The requirement to present a completed project that was nominated and supported by the hospital Board of Directors and EMT gave influence and a voice to the project groups.

#### 3.1.4. Sense-Making

The deployment of LSS in conjunction with person-centred care commenced in the hospital in 2017. The following practical aspects of deployment were also put in place to support the above principles. All staff members were included in invites to attend training events. LSS training events were advertised through hospital-wide newsletters, email groups, team meetings, etc. Every staff member was invited to attend White Belt training. Staff from all disciplines and grades attended White Belt training together; there was no specific training for members of the EMT. This supported the hospital’s values of removing barriers between senior managers and staff directly involved in patient care as well as encouraging collaboration across teams/moving from a siloed approach to process improvement. White Belt training had to be completed before moving on to Green Belt training. Academic institution requirements were also noted. Once a staff member was assigned a place at a training event, they agreed to participate actively in the training event. To encourage collaboration, training events were arranged with team members from different departments and at different levels of seniority.

To ensure a whole system approach to improvement, each staff member applying for Green or Black Belt training was asked to submit a project charter as part of their application. Members of the EMT and quality and patient safety staff committed time to potential students to co-design project suggestions and project charters. This ensured that projects were aligned to the strategic goals and direction of the organisation. From a staff perspective, this also demonstrated the EMT and senior management commitment to their improvement project. This commitment was also demonstrated in practice. To assist with staff being released for improvement work, each application required approval from the staff member’s line manager—to ensure cover was in place for the staff member’s improvement leave as required. The first White Belt course was delivered in May 2017. Attendees included the CEO, a nurse specialist, a procurement operative, a physiotherapist, a healthcare assistant, and a patient services administrator. The ETWG had achieved a very important goal—the training event was accessible to all and had served to show that hierarchy was not going to be a barrier to improvement [67].

Following the implementation of White Belt training events, the organisation was ready to submit applications for Green Belt training commencing September 2017. For the candidates proceeding to Green Belt training, the organisation and candidates hoped that this would empower “middles” to lead process improvement by giving them the skills to integrate the needs and requirements of management with the potential and skills of the frontline staff [60]. The first Black Belt training programme was completed in November 2020, delivering advanced knowledge on LSS in healthcare. This also delivered the very significant milestone of the hospital being able to deliver White Belt training internally.

Each LSS training event resulted in specific deliverables. At the Black Belt/Green Belt level, this was the completion of process improvement projects with a tangible impact on the strategic goals of the organisation. At the White Belt level, a network of staff familiar with LSS tools was developed who could assist Black and Green Belts to achieve project goals. Every staff member in the hospital has a role to play in quality and patient safety. The accessibility of LSS to all staff created an avenue for all staff to learn and become actively involved in patient safety activities. Combining a person-centred approach and stakeholder engagement methodology, a shared purpose approach has emerged in the LSS projects to date. The project teams formed and refined the project goals and took a shared responsibility with key stakeholders to see projects through to completion.

### 3.2. Case Study Synthesis

The importance of taking a socio-technical systems approach to whole system change that focuses on the four domains of culture, system, action, and sense-making was stressed in the Introduction [33] as an important approach to move forward the lack of traction on quality and patient safety improvement that has afflicted healthcare over the last 20 years [1,2].

The results of this case study are now discussed with these four domains in mind. At the outset, the organisation required increased knowledge and skills in person-centred process improvement to help staff provide a sustainable workforce that could engage with and support organisation expansion and development. The person-centred implementation of LSS in the organisation has resulted in the emergence of a task-based culture that focuses on involving the right people with the right resources to complete improvements [61]. The unifying power of the group is in their approach to the project—a commonality in structuring the project utilising LSS tools based on the principles of collaboration, inclusion, and participation [27]. These principles allow staff who have completed Green and Black Belt training to support process improvement outside of their usual areas of work—moving away from silo-based improvement or ‘projectitis’ and to more of a system-wide approach to change. LSS graduates from one area are supporting improvement in another. This enables sharing of knowledge and skills, the building up of organisational trust, systemic learning at both a tacit [63] and explicit level, and the provision of support to system-wide improvement. Interdependencies between projects and areas are noted and a systems view emerges. Staff from patient services supported improvement projects in theatre procurement and graduates from physiotherapy supported projects in information technology/education planning. Investing time and energy to allow staff to do this can be a challenge in a busy acute hospital. By employing the principles of stakeholder engagement promoted by LSS—seeking to understand and giving voice, but also ensuring improvement sessions were well structured with identifiable deliverables, staff were happy to dedicate time to achieve the desired outcome and the organisation supported this.

Study leave was approved before Green Belt and Black Belt training and education commenced. A support network for covering staff was agreed upon. The clear message of support from the Board and EMT removed concerns regarding financial and study leave support. More challenging was facilitating stakeholder engagement/data collection sessions. Teams had to be mindful to meet their stakeholders at times and venues that suited. Additionally, hugely important was the need to reassure stakeholders that the teams sought to understand processes and challenges and seek solutions. The purpose of a LSS project was never to examine or find fault with the person—94% of the problems are caused by the system and 6% by the individual [68].

In terms of the development of a long-term sustainable team that can support hospital development and expansion, the hospital has moved through forming, storming, and norming and is currently progressing to performing [69]. D’Andrematteo (2015) [70] called for further investigation into the organisation-wide success and weakness of LSS. In this system-wide implementation of LSS underpinned by a person-centred approach, the hospital has achieved an organisation-wide approach to improvement involving staff from all specialities and levels of seniority.

Benefits and challenges involving roles within the improvement team were noted. The involvement of clinicians in healthcare improvement is central to system change [71]. There was great support from clinicians throughout—from practical support given by the Orthopaedic Consultants and Anaesthetist in implementing Day Case Anterior Cruciate Ligament surgery to the “external” process view offered by the Speech and Language Therapist to theatre procurement and stock management [55]. Each LSS project is based on the collaboration of team members from a combination of medical, nursing, HSCP, and management/administrative backgrounds [72].

Clinicians are trained to make quick decisions to address an evolving presentation in a patient. The temptation to start a process improvement with “I know the solution—we just have to …..” was something that a lot of staff had to learn to avoid. Process owners within teams also had to learn to allow others the authority to examine processes and facilitate stakeholder engagement and data collection—in some cases acknowledging that team members from outside the process were better placed to complete these activities—as they approached them with “fresh eyes”. This supports a culture where all staff members have psychological safety [67] and feel able to speak up for important issues such as quality and safety of patient care [68]. Psychological safety is an essential component of achieving JCI accreditation [40]. It helps healthcare move on a journey towards high reliability [1] and to building organisational resilience [73]. The management is also learning to distribute power and knowledge and acknowledge the expertise and insights of others. There is less emphasis on the positional role and traditional authority [74,75].

LSS is now the method of choice used for improving processes. LSS is also used to present improvements as part of JCI accreditation. The organisation completes JCI accreditation every three years. As part of this accreditation, the hospital reports on key performance indicators, including length of stay and readmission rates, and quality improvement projects around these indicators. Please see Table 8.

From 2019, these projects have been completed using the LSS methodology. The hospital first achieved JCI accreditation in 2007 and has been re-accredited every three years since then—most recently in 2019. Continuing to achieve re-accreditation requires continuing improvement as well as a commitment to quality and safety of care, including the International Patient Safety Goals.

In addition to the projects described above and as a reflection of the maturing of a LSS culture in the organisation, the LSS methodology has now been adopted as the process improvement method of choice in the organisation. Green and Black Belt projects, as mentioned above, have led to legacy projects outside of the academic structure.

As the number of staff familiar with the LSS approach increases in the organisation, the use of various methods, tools, and strategies has become commonplace. For example, when planning a new or changed service, first thoughts are always to align with the strategic objectives of the organisation, followed by using LSS tools such as process mapping to understand how the service currently runs (AS IS mapping) and to identify how the service will run (TO BE mapping). When analysing potential risks associated with changing a process, a Failure Modes Effect Analysis (FMEA) is completed as standard—this is of particular benefit when preparing for JCI accreditation as it is a tool that JCI commonly requests as part of their accreditation of quality and safety improvement in the hospital.

The CUBE STS analysis framework as further developed in the Access Risk Knowledge (ARK) Platform addresses questions of value in terms of the projected gain and the actual gain of the change achieved [28,66]. In Table 7 the expected outcome and the actual outcome achieved are presented for each individual project. Improvements also occurred outside of these projected outcomes, for example, improvements related to operation notes also improved patient safety and created a template for the transference of further documents to the patient electronic record—without having to seek external consultancy advice. Value can also be seen by stakeholder satisfaction and improved patient care. Examples of stakeholder satisfaction include:

*“The novelty, of actually being able to read the handwriting and understand the detail of the surgery, is brilliant!”*

*“It’s so easy to use”,*

*“With the help of the templates, I can complete my Op note in minutes”*

*“It’s saving me so much time!”*

*“Love the layout, it’s so easy to read”*

Harder to estimate is overall Return on Investment (ROI). Four years into the deployment, ROI can be estimated by savings made related to improvement projects. Each of the seven studies reported on here achieved outcomes that can be quantified separately, e.g., projects involving theatre stock have led to a 91% reduction or EUR 24,769 in the value of out-of-date stock and a 45% reduction in nursing stock preparation time (releasing that nursing time to caring for patients) [51,55]. Projects involving patient flow, such as improving the pathway for patients attending Anterior Cruciate Ligament reconstruction, have resulted in an additional 24.6 bed days annually in the organisation [56]. This implementation was funded within the existing postgraduate education and training budget. Analysis of staff retention and progression is complicated due to many changing circumstances resulting from the COVID-19 pandemic. Of the 32 staff who have completed Lean Six Sigma practitioner training, 25 (78%) remain and are progressing to new roles in the organisation. Further analysis of the 21% of staff trained who have left the organisation is required to identify motivating factors behind the staff member’s decision to change.

Another ROI was the ability to continue White Belt training with in-house resources, meaning the cost of continuing LSS training in the organisation reduced significantly in 2020. Perhaps a mark of leadership satisfaction with the LSS programme was that rather than allocating those savings to another area, the savings were ploughed back into LSS training and education—supporting further Green Belt and Black Belt training.

## 4. Discussion

The case study synthesis, using the CUBE domains of culture, action, system functioning, and sense-making combined with Oshry’s OSF, has enabled us to answer the question of whether or not these elements combined to create agency for change at the organisational level of the hospital. The case study demonstrates evidence of whole system improvement; projects involving staff from all specialities and levels of seniority are linked to each other and to the strategic objectives of the organisation, and learnings from first-generation projects have been passed to second and third-generation process improvements.

The question of whole system change is difficult, however. There is little agreement in the literature on what constitutes ‘whole system’ change, which speaks to the origins of this special issue. This case study has taken the approach that the design of an effective agency of complex and socio-technical system change requires both an understanding of socio-technical systems and the engineering of their development [28] and takes some reflection on our role as actors within the system [47,48].

Flynn et al. (2019) [77] completed a realist evaluation to identify *contexts* and *mechanisms* that enabled and hindered implementation and had an effect on the *outcome* of sustainability of what was meant to be a whole system Lean intervention in a pediatric healthcare setting (CMOs). This intervention was noted as being the ‘largest Lean transformation in the world’ [78]. While Flynn et al.’s evaluation focused on the outcome of sustainability, the framework could still be used to assess whether the hospital intervention reported here did have an impact at a systems level. The CMOs from Flynn et al.’s work are thus presented here along with a response from the synthesis of evidence in this case study.

CMO1: The early stages of Lean’s implementation were funded, mandated, and top-down in nature (C), driven by an external consultancy firm that initially focused on training senior leadership (C). Frontline staff did not feel involved in Lean changes, and they felt pressured to adopt Lean (M). The Lean language used did not make sense to staff (M). Training failed to demonstrate a connection between Lean and healthcare.

In this case study, it can be seen that an approach to whole system improvement was co-designed from within the system by a team of staff (ETWG) in conjunction with the Board of Directors and EMT. A partnership approach was developed with the UCD Lean Academy who are a team of former and current healthcare workers who have adopted LSS for healthcare staff. The training used and examples given were based in the Irish healthcare settings. The UCD Lean Academy has committed to supporting healthcare teams publish their research to add to the international evidence base [12,13,14,15,79]. Materials from these cases studies were used to support the training.

CMO2: The complexity and dynamic nature of healthcare (C) were perceived as incongruent with the nature of Lean. The translation of Lean to patient care did not make sense for many staff and Lean efforts felt impersonal. Lean training failed to make the connection between Lean and healthcare clear for staff (M) and the early stages of implementation led by the consultancy company failed to customise Lean to the local context. This triggered pitfalls to the success of Lean, such as feelings of disconnection and negative perceptions of Lean (M), resulting in resistance to and a lack of support for Lean continuation (O).

In this case study, it was seen that LSS process improvements were designed and led by organisational staff from the outset with support from staff from the Lean Academy. Organisation stakeholders met with their colleagues rather than with an external consultant. This enabled a shared approach to understanding the challenges, the joint consideration of solutions, and an acknowledgement of previous efforts at improvement made in the past, rather than a suggestion of “just do it” solutions.

CMO3: Lean was implemented in areas that experience constant change (C), early stages of implementation involved multiple Lean events for training purposes (C), and frontline staff felt overwhelmed from the constant change, they were unsure what changes were due to Lean, and felt that Lean was the latest fad (M). This led to negative perceptions of Lean, resistance, and a lack of support by frontline staff (O).

As a relatively young organisation, staff are accustomed to change and progression with short lead-in times. In this case study, it was evident that rather than change being seen as a challenge, the use of LSS and data-driven solution design allowed team members to participate actively in change and take ownership and credit when solutions were found.

CMO4: The contract of the external consultancy leading Lean’s implementation ended (C), placing the continuation of Lean on internal senior leaders and unit managers (C). This led to a process of customisation of Lean to the local context through a variety of ways. This customisation of Lean and shift in implementation triggered positive and negative responses from frontline staff, unit managers, and senior leaders (M). As a result, only some Lean efforts became embedded. However, there was variation and a discrepancy between senior leaders and unit managers compared with frontline staff on perceptions of how embedded Lean efforts were (O).

In this case study, it was seen that the hospital system was committed to building up in-house expertise from the beginning via the training of White, Green, and Black Belts who would reinvest in the system and train further White Belts.

CMO5: The context of early stages of implementation (C) failed to trigger sense-making processes necessary for staff to understand Lean and potentially engage with and begin to embed Lean into their practices (O). Shared values were evident between Lean principles and staff professional values as healthcare providers. However, value congruency without clear sense-making processes resulted in a lack of adoption of Lean behaviours as part of normalised frontline practices. Sense-making processes were hindered by a failure of initial Lean training efforts to translate the principles of Lean into the context of healthcare that would resonate with staff (M). Lean language and the lack of staff involvement in Lean changes also hindered sense-making processes and feelings of engagement. This resulted in negative perceptions of Lean, a lack of buy-in, and a lack of support for the continuation of Lean from frontline staff (O).

In this case study, it can be seen that there was a focus on sense-making from the outset. One learning from the LSS deployment to date is the need to explore and understand the pain/challenge from all perspectives from the outset.

### Strengths and Limitations

The strengths of taking a case study approach are that it allows us to attempt to answer complex questions by triangulating different data from different sources [43]. Internal consistency was increased by collecting data from multiple sources and by using different types and sources of data. Reliability was aided by transparency in terms of outlining the questions and processes of synthesis [80].

A criticism, however, of this study could be that only one author (MEW) was outside of the process as it was happening. However, there is also a strength in combining insider insights on change and using the rigour of a STS analytic framework such as the CUBE combined with Oshry’s Organic Systems Framework to approach the case study.

A further point to be acknowledged is that this case study reports on the system that was one hospital. This is the strength of the case study approach and helps us give importance to and answer questions on topics in their own right. However, as noted above, whole system change is complex and there may be other factors at play when we consider a ‘systems-of-systems’ approach and acknowledge the wider impact of societal, legislative, political, and other factors on that system. As Flynn et al. note in this special issue [81], there is growing traction for the need to look at what has been termed ‘learning health systems, which are dynamic ecosystems where scientific, social, technological, policy, legal, and ethical dimensions are aligned to enable continuous learning and improvement to be embedded across the system [82]. COVID-19 has also taught us a great deal about the importance of taking a ‘systems-of-systems’ approach in healthcare and there are further lessons to be learned from this [83].

## 5. Conclusions

There are strengths and limits to the case study approach; however, we hope here, guided by an STS approach, to add to the body of literature on what would constitute whole system improvement in healthcare. Recognising the organisation’s *culture*, aligning complex *system* functionality requirements and the ability to *activate* these requirements to deliver concrete outcomes, and developing a shared understanding or sense-making of future goals aligned with embedding a person-centred approach to whole system improvement have synergised in a way that credibly addresses what it takes to change a whole system. Through the growing organisation-wide knowledge of the LSS approach and methods underpinned by person-centredness [27], the hospital is creating an increasing network of those who, in Oshry’s terms, “can”, “know”, and “want” to continuously strive for improvement in the quality and safety of patient care in the organisation [60]. This case study highlights achievements to date. The organisation will continue to grow and develop process improvement with a growing network of staff to support this important work. The STSA CUBE framework and Oshry’s OS framework were used here retrospectively to assess an intervention but could also be used prospectively to help healthcare organisations develop approaches to whole system improvement. Future areas of development for this organisation and to promote the sustainability of LSS and person-centred care include: (1) assessing the impact of LSS/person-centred process improvement through a stakeholder survey as well as the recording of formal project outputs; (2) disseminating and celebrating achievements internally and externally; and (3) continuing to reinvest in training and education to ensure leaders and process improvers remain equipped with skills and knowledge in this constantly evolving field.

## Figures and Tables

**Table 1:** High-level questions are derived from the STSA CUBE [46].

	Culture	Functioning System	Action	Sense-Making
Goals	What are the cultural values of people working in the organisation?	What are the system goals?	What are the key outcomes of the current situation and how are they measured?	What are the objectives of key stakeholders?
Process	What are the norms of behaviour and everyday practice?	What are the key tasks and activities, and how effective is the current sequence?	What data and indicators are used to assess performance?	What is the quality of the tasks and activities being carried out?
Social Relations	What different professional groups/subcultures work together?	What are the key roles and relationships (working with, reporting to)?	How are roles and relationships documented and assessed?	What is the quality of leadership and collaboration?
Information and knowledge	Is there a shared understanding of what to do and how the system works?	Can we describe the flow of information that links people to their activity?	How is the quality of information, knowledge, and information flow measured?	What is the quality and flow of information like, with regards to enabling informed action?

**Table 2:** Reflective questions derived from Oshry’s Organic Systems Framework [48,49].

	Tops (Executive)	Middles (Middle Managers)	Frontline (Administration, Clinical)	Customers/Clients/Patients/Insurance
Question	To what extent and how were leadership and authority distributed and supported?	To what extent and how were they empowered to act to design and implement (agency)?	To what extent and how were they persuaded to engage and become involved?	To what extent and how did they enter into a working partnership?

**Table 3:** The organisation’s strategic goals.

Leadership Goal	JCI Chapter	Improvement Required/Target
The quality and safety of patient care	Patient safety goals and all JCI chapters	Maintenance of JCI accreditation throughout the whole system change process and in particular in relation to the six International Patient Safety Goals: (i) Identify patients correctly; (ii) Improve effective communication; (iii) Improve the safety of high-alert medication; (iv) Ensure safe surgery; (v) Reduce the risk of healthcare-associated infections, and (vi) Reduce the risk of patient harm resulting from falls.
Information Technology Enhancing Safer Patient Care	GLD/MOI	Organisational goal to evolve to a fully paperless/electronic patient record
Improve Patient Flow	ASC, AOP, COP	Optimise patient flow through ensuring correct resources are available and utilised for each step of the patient journey
Care of High-Risk Patient	AOP, COP, FMS, GLD, SQE	Deliver optimum care to the high-risk patient through early identification, availability of specialist clinicians, and adaptation of best practice guidelines.

**Table 4:** System and action table.

Leadership Goal	JCI Chapter	Project Title	Process to Improve	Expected Outcome
The quality and safety of patient care	All	Support and oversee Lean Six Sigma process improvements		Visibility on all projects and Patient Safety and Quality Improvement aspects of them; linking of project goals to the JCI accreditation process and International Patient Safety Goals
Information Technology Enhancing Safer Patient Care	GLD/MOI	Operation Note Transformation: the application of Lean Six Sigma to improve the process of documenting the Operation Note in a Private Hospital Setting [50]	Process for documentation of operation notes	100% of operation notes completed electronically
Improve Patient Flow	ASC, AOP, COP	We’ve Got your Back: improve scheduling of patients for spinal surgery	Time frame for confirmation of spinal surgery	Time for admittance for spinal surgery confirmed 72 h pre-surgery
		Book Right first time—Redesigning the Process for Scheduling Elective Orthopaedic Surgery: A Combined Lean Six Sigma and Person-Centred Approach [52]	Process for scheduling elective orthopaedic surgery	100% of elective orthopaedic surgerie scheduled within 48 h of consultant appointment
		The Use of Lean Six Sigma Methodology in Reducing Length of Stay and Improving Patient Pathway in Anterior Cruciate Ligament (ACL) Reconstruction Surgery [56]	Length of Stay for Anterior Cruciate Ligament patients	Length of stay of <24 h for patients admitted for ACL surgery
		The Use of Lean Six Sigma for Improving Availability and Access to Emergency Department data to facilitate patient flow [54]	Data availability regarding patient flow through Emergency Department	Data regarding ED patient flow are available to stakeholders when required
		A Heartbeat in Time—use of Lean Six Sigma to improve patient flow in Cardiology Department	Patient flow through Cardiology	Reduce the length of stay for Cardiology patients
Care of High-Risk Patient	AOP, COP, FMS, GLD, SQE	Lean Six Sigma Redesign of a Process for Healthcare Mandatory Education in Basic Life Support—A Pilot Study [53]	Provision of mandatory training	Review process for accessing Basic Life Support training with a focus on optimising delivery methods
		Using Lean Six Sigma to redesign the Supply Chain to the Operating Room Department of Private Hospital to Reduce Associated Costs and Release Nursing Time to Care [55]	Preparing stock required for surgery	Standardise process for stock handling. Reduce the value of stock going out of date by 50%. Optimise theatre storage areas.
		Releasing Operating Room Nursing Time to Care through the Reduction of Surgical Case Preparation Time: A Lean Six Sigma Pilot Study [51]	Preparing specialist equipment required for surgery	Reduce preparation time for surgical cases to release nursing time to care for patients
		Releasing Nursing Time to Care—Use of Lean Six Sigma to redesign Health Care Assistant training and skills	Training and tasks allocated to Health Care Assistants	Reduce non-value-added activities in a nursing shift by standardising the role of the Health Care Assistant and developing the role to support the care of a patient

**Table 5:** Education and Training Working Group.

Position	Responsibility	Role in the Working Group	Stakeholder Engagement
Director of Human Resources (HR)	Responsible for supporting staff recruitment, retention, training needs analysis, and performance review	Expert knowledge of factors impacting staff recruitment, retention, and progression	Administration functions include patient services, finance, marketing, and Human Resources
Director of Nursing (DON)	Responsible for delivery of nursing care in the organisation	Expert knowledge of progression planning and career pathways of team members with leadership and innovation skills	All nursing staff
Chief Operations Officer (COO)	Responsible for oversight of organisation operations including Quality, Patient Safety, and Innovation	Expert knowledge of strategic goals and organisational targets.	Quality and Patient Safety
UCD Beacon Academy manager	Responsible for supporting postgraduate training and research opportunities	The direct link with third-level education facilities and wider healthcare education groups. Expertise in externally available programmes and how they may be implemented in the organisation	Allied Health/Health and Social Care Professionals (HSCPs) and non-consultant hospital doctors (NCHDs)

**Table 6:** Outputs from stakeholder engagement sessions.

	Quality Improvement, Leadership, Management	Access	Project Delivery	Academic Qualification
Focus group themes	Process improvement methodologies	Getting time to do education is hard	We start so many things but do not finish	Commitment to academic qualification means extra effort
	How to get the best out of a team	Education is for the younger staff	We are never asked to get involved in projects	Qualifications to suit all levels
	I have lots of ideas but I cannot bring about change	I have done a Master’s, I do not need to do any more	There are only two of my discipline in the organisation—we are asked to get involved in everything	Accessible to all staff
	I am too junior to be involved in improvement projects	Flexible in delivery	Project management skills	A clear outline of commitment is required.
	How to measure outputs and continuing improvement	Accessible to all (Bachelor’s Degree not required)	Organisation/system-wide approach	
		Part-time	Person-centred and interdisciplinary working	

**Table 7:** LSS projects delivered through collaborative, inclusive, and participative working.

Leadership Goal	JCI Chapter	Project Title	Process to Improve	Team Members Involved	Actual Outcome
The quality and safety of patient care	All	Central oversight overall projects		EMT, Lean Six Sigma practitioners.	Visibility on all projects, including goals, supporting process improvement, and monitoring outcomes.
Information Technology Enhancing Safer Patient Care	GLD/MOI	Operation Note Transformation: The application of Lean Six Sigma to improve the process of documenting the Operation Note in a Private Hospital Setting [50]	Process for documentation of operation notes	IT project manager, Developer, Head of Surgery, Theatre Nurse Manager	100% of operation notes completed electronically
Improve Patient Flow	ASC, AOP, COP	We’ve Got your back: improve scheduling of patients for spinal surgery	Time frame for confirmation of spinal surgery	Administrator, patient services, clinical nurse manager, surgical day unit	Time for admittance for spinal surgery confirmed 72 h pre-surgery
		Book Right first time—Redesigning the Process for Scheduling Elective Orthopaedic Surgery: A Combined Lean Six Sigma and Person-Centred Approach [52]	Process for scheduling elective orthopaedic surgery	Physiotherapy manager, patient services staff member, nurse	100% of elective orthopaedic surgeries scheduled within 48 h of consultant appointment
		The Use of Lean Six Sigma Methodology in Reducing Length of Stay and Improving Patient Pathway in Anterior Cruciate Ligament Reconstruction Surgery [56]	Length of Stay for Anterior Cruciate Ligament patients	Physiotherapist, Data Analyst, Project manager	Length of stay of patients admitted for ACL surgery reduced by 15.9 h
		The Use of Lean Six Sigma for Improving Availability and Access to Emergency Department data to facilitate patient flow [54]	Data availability regarding patient flow through Emergency Department	Physiotherapy Manager, Developer, Emergency Department manager	Data regarding ED patient flow available to stakeholders when required. 495 min of nursing time per day released to patient care.
		A Heartbeat in Time—Use of Lean Six Sigma to improve patient flow in Cardiology Department	Patient flow through Cardiology	Clinical nurse manager, Bed manager, medical records staff member, patient services staff member, patient accounts team member	17% improvement in the number of patients discharged by the target time of 10 am.
Care of High-Risk Patient	AOP, COP, FMS, GLD, SQE	Lean Six Sigma Redesign of a Process for Healthcare Mandatory Education in Basic Life Support—A Pilot Study [53]	Provision of Mandatory training	Clinical nurse educator, Emergency Department manager, Quality and patient safety analyst, administrator, patient services team member	50% increase in capacity to deliver Basic Life Support with the same resources. Saving of EUR 5500 per annum
		Using Lean Six Sigma to redesign the Supply Chain to the Operating Room Department of Private Hospital to Reduce Associated Costs and Release Nursing Time to Care [55]	Preparing stock required for surgery	Procurement manager, speech and language therapist, quality and patient safety analyst	Reduction in the value of stock going out of date by 91% or EUR 24,769 Reduction in time spent preparing stock for procedures by 45%
		Releasing Operating Room Nursing Time to Care through the Reduction of Surgical Case Preparation Time: A Lean Six Sigma Pilot Study [51]	Preparing specialist equipment required for surgery	Head of Radiology, physiotherapist, administrator, Theatre Nurse Manager, procurement team member	55% reduction in time spent preparing materials for surgical cases.
		Releasing Nursing Time to Care—Use of Lean Six Sigma to redesign Health Care Assistant training and skills	Training and tasks allocated to Health Care Assistants	Head of Radiotherapy, Administrator, procurement team member	Reduction of non-value-added activities in a nursing shift by 95 min per nursing shift and 84 min in a Health Care assistant shift.

**Table 8:** Hospital leadership goals and key performance indicators.

Leadership Goal	Key Performance Indicator
Improve patient flow	Length of stay
	Readmission rate after 30 days
Improve the care of the high-risk patient	International Patient Safety Goals
Compliance with International Patient Safety Goals	Quality Improvement Project and Key Performance Indicator linked to each Patient Safety Goal
	Number of Patient Identification Errors
	Clinical handovers completed in compliance with the ISBAR communication tool
	Number of Medication safety events
	Compliance with WHO surgical safety checklist/Time out compliance [76]
	Hand Hygiene compliance/surgical site infection rate
	Falls rate
Information technology enhancing patient care	Chart audit of compliance with documentation/healthcare records guidelines

## Data Availability

Not applicable.
